# Supplementary material for: Enhancing the efficacy of B7-H3-targeting antibody-drug conjugate DS-7300a in TP53-deficient tumors by inducing ferroptosis
Source: Oncogene. 2026 Jul 27;45(36):3753–64. doi: 10.1038/s41388-026-03921-7 (PMC13529587; doi:10.1038/s41388-026-03921-7)
Supplement: Supplementary file 1 [file 41388_2026_3921_MOESM1_ESM.pdf]

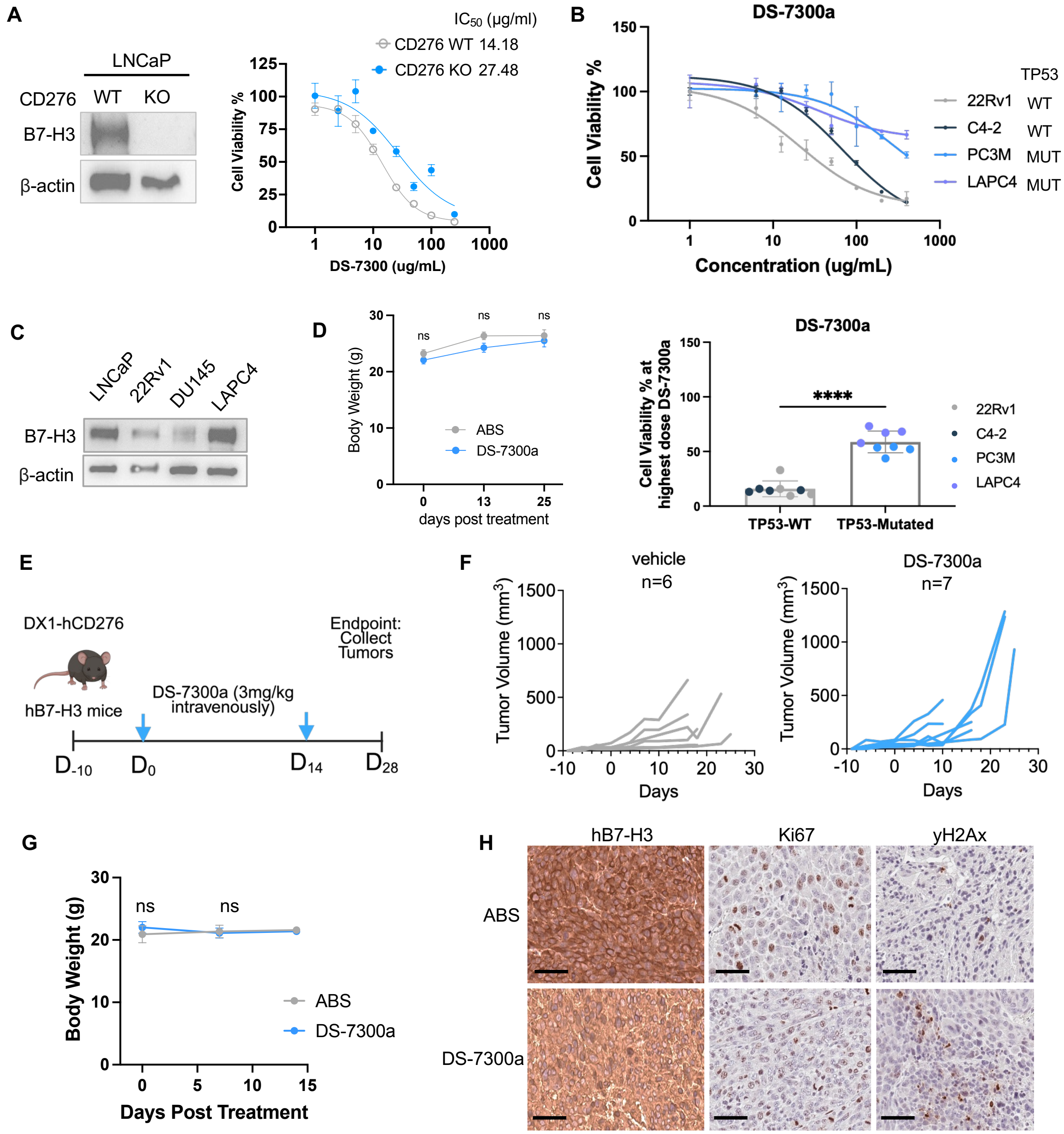

Supplementary Figure 1. TP53 defects confer resistance to DS-7300a, a B7-H3-targeting ADC.

A. Western blot of B7-H3 and dose-response assays present the sensitivity of CD276-wildtype (WT) and CD276-knockout (KO) LNCaP cells to DS-7300a. IC$_{50}$ values are shown. **B.** IC$_{50}$ determination of DS-7300a in PCa cell lines containing wildtype or mutated *TP53*. Cells were treated with the indicated concentrations of DS-7300a for 96 hours in all experiments with at least three replicates. Dose-response curves are presented at the top. The bottom bar plot shows the % cell viability of each cell line 96 hours after DS-7300a treatment at the concentration of 400 µg/ml. **C**. Western blot analysis of B7-H3 protein in various PCa cell lines containing various TP53 status. **D.** SCID mice bearing LNCaP isogeneic tumors were treated with ABS or DS-7300a (0.5 mg/kg). Body weight of mice over the course of treatment were documented. **E-H.** 2×10^6 DX1-hCD276 cells were subcutaneously injected into both flanks of male B-hB7-H3 mice, followed by treatment of ABS vehicle control or DS-7300a (3 mg/kg; i.v., biweekly, twice). (**E**) Schematic of experimental design using humanized B7-H3 models. Tumor growth curves of individual DX1-hCD276 tumors (**F**) and body weights of mice (**G**) treated with ABS or DS-7300a (3 mg/kg). (**H**) Immunohistochemistry analysis of hB7H3, Ki67 and DNA damage markers in DX1-hCD276 tumors after treatment. Scale bar = 50um. Data represent the mean ± standard deviation of triplicates or otherwise stated. *P* values determined by unpaired two-tailed t test; *P < 0.05; **P < 0.01; ***P < 0.001; ****P < 0.0001; ns, not significant. **E** was created in BioRender. Leo, J. (2026) https://BioRender.com/daimkj1.

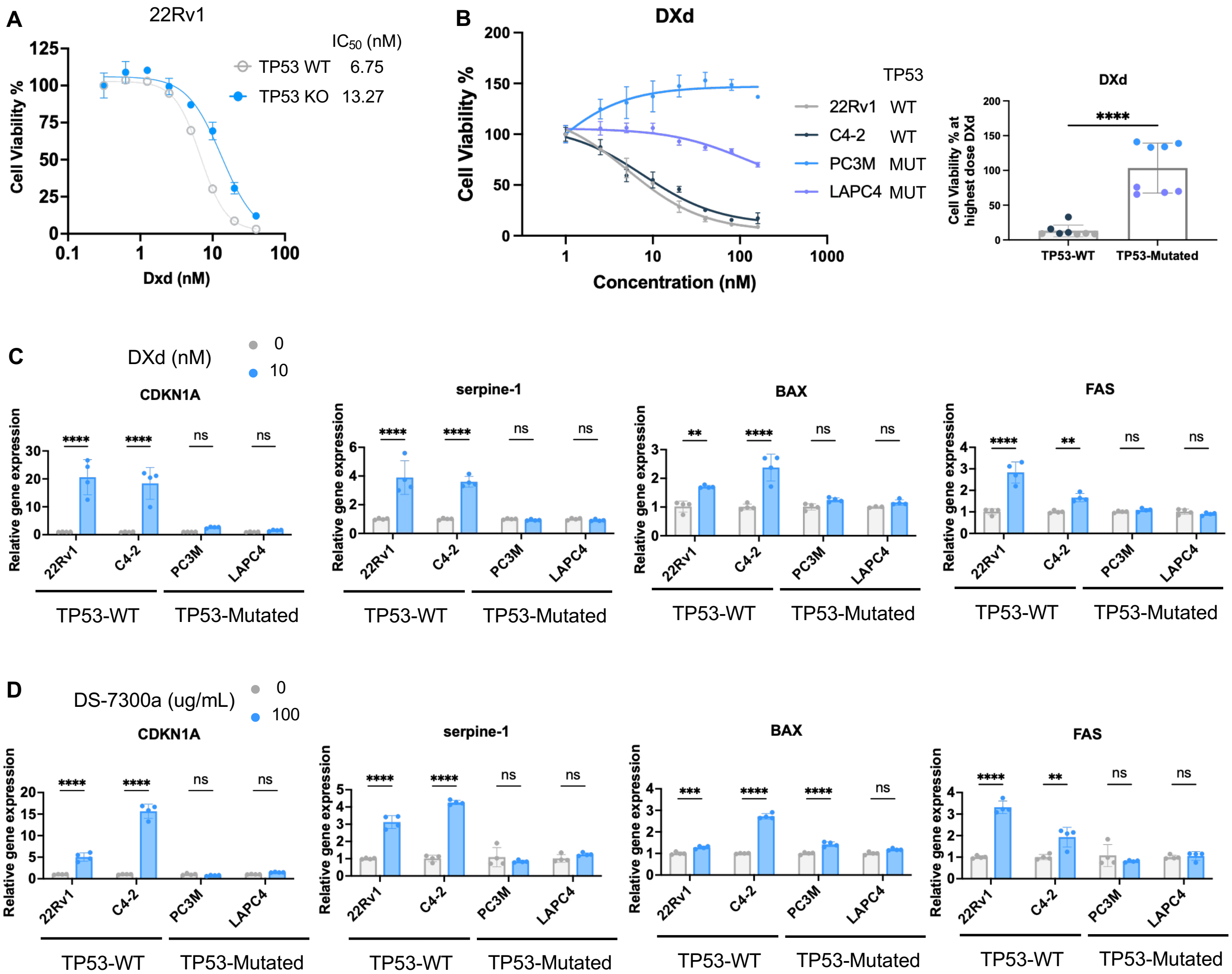

Supplementary Figure 2. DS-7300a and its payload DXd triggers apoptosis and senescence via activation of p53 signaling in TP53-intact prostate cancer cell lines

A. Dose-response curves and IC_{50} of DXd in 22Rv1 with or without TP53 KO. **B.** IC_{50} determination of DXd in diverse PCa cell lines containing wildtype or mutated *TP53*. Cells were treated with the indicated concentrations of DXd for 48 hours in all experiments with at least three replicates. Dose-response curves are presented at the left. The right bar plot exhibits % of cell viability of each cell line 96 hours after DXd treatment at the concentration of 160nM. **C-D.** mRNA expression levels of TP53 direct target genes involved in senescence and apoptosis in indicated PCa cells after 48h DXd treatment (C) or 96h DS-7300a treatment (D), determined by qPCR. Data represent the mean ± standard deviation of triplicates or quadruplicates. IC_{50} values were calculated using GraphPad Prism version 10.4.1. *P* values determined by two-way ANOVA analysis (C-D); *P < 0.05; **P < 0.01; ***P < 0.001; ****P < 0.0001; ns, not significant.

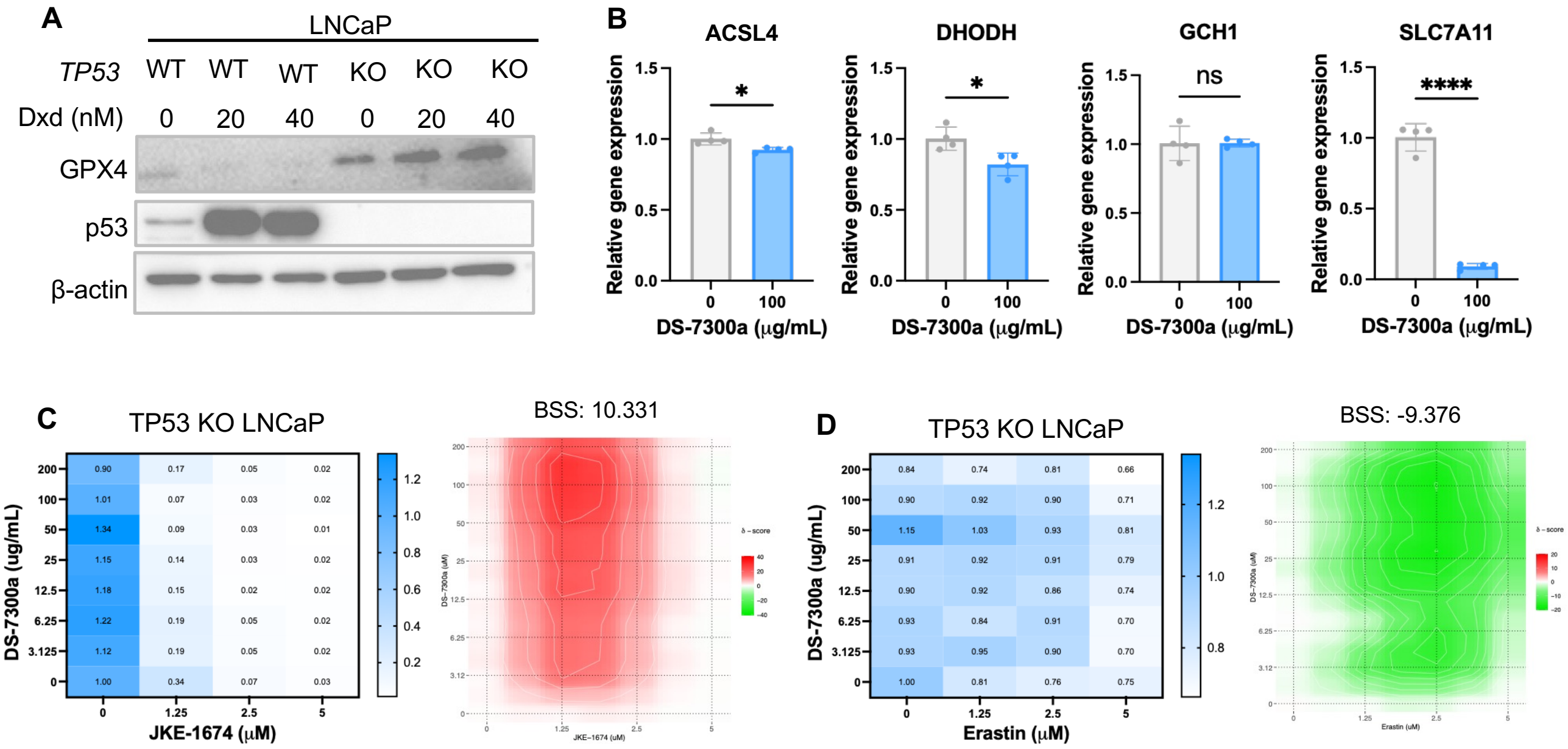

Supplementary Figure 3. Ferroptosis inducer targeting GPX4 enhances the anti-tumor effects of DS-7300a in TP53-deficient tumors.

A. Western blot analysis of GPX4 and p53 in TP53-WT and TP53-KO LNCaP cells after DXd treatment. **B.** mRNA expression of ferroptotic-associated genes in TP53-KO LNCaP cells treated with DS-7300a. Data represent the mean ± standard deviation of quadruplicates. **C.** Dose-response matrix (left) and drug interaction plots (right) revealing the synergistic effects of DS-7300a and JKE-1674 in TP53-KO LNCaP cells. **D.** Dose-response matrix (left) and drug interaction plots (right) revealing the antagonistic effects of DS-7300a and Erastin in TP53-KO LNCaP cells. Drug interaction landscapes and Bliss synergy score (BSS) were generated using SynergyFinder. *P* values determined by unpaired, two-tailed t test; *P < 0.05; ****P < 0.0001; ns, not significant.

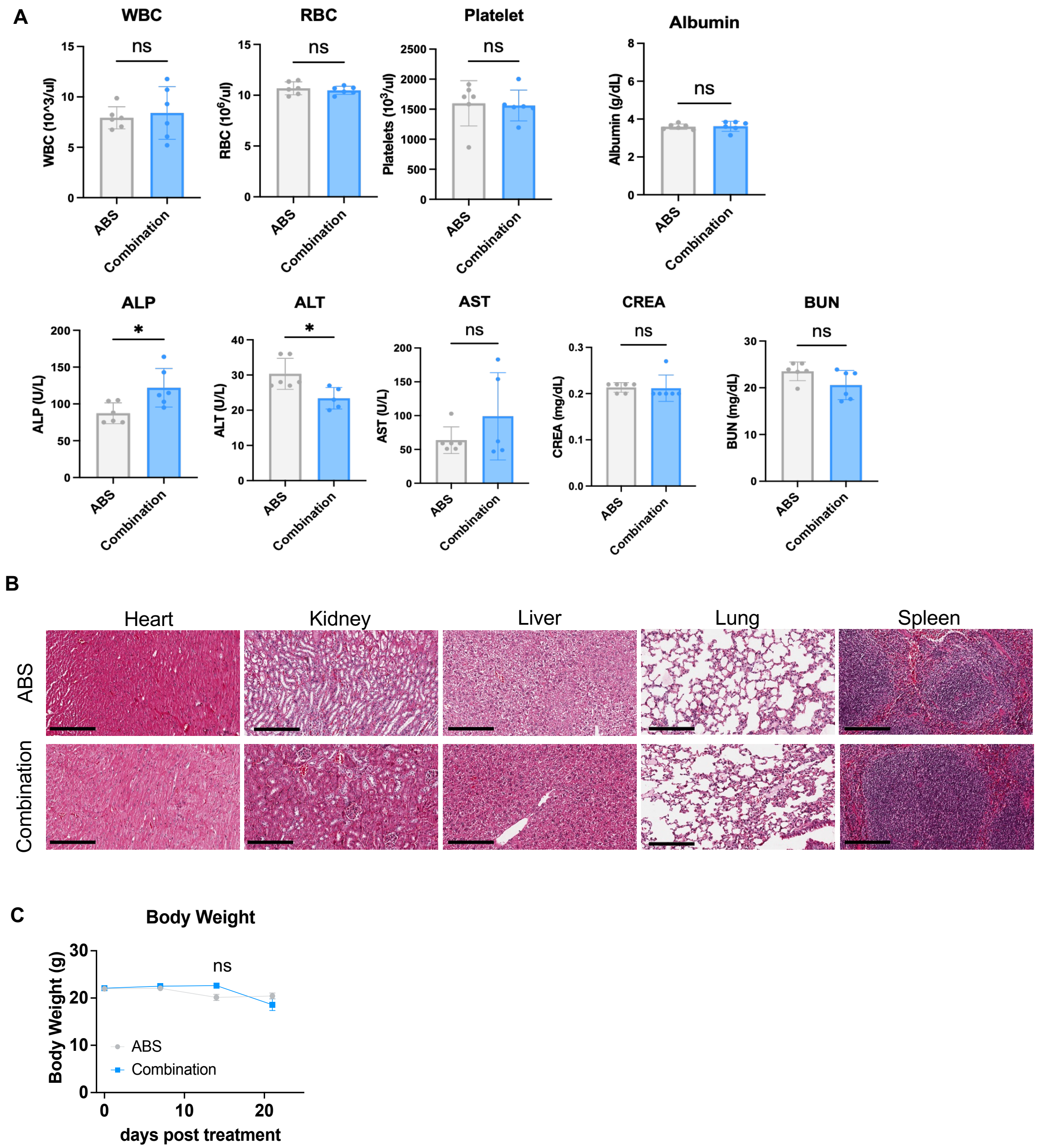

Supplementary Figure 4. The combination of DS-7300a and ferroptosis inducer JKE-1674 showed little toxicity.

A-B. Tumor-bearing B-hB7-H3 mice were treated with ABS (vehicle control) or the combination of DS-7300a (2mg/kg intravenously) and JKE-1674 (10mg/kg oral gavage), followed by blood test (**A**) and H&E staining of indicated organs (**B,** scale bar = 200um). Standard blood panels include white blood cell (WBC), red blood cell (RBC), and platelet counts. Albumin, Alkaline phosphatase (ALP), alanine aminotransferase (ALT), and aspartate aminotransferase (AST) levels were screened for assessing hepatotoxicity; while creatinine (CREA) and blood urea nitrogen (BUN) levels were screened for assessing nephrotoxicity. **C**. Body weight of tumor-bearing SCID mice received ABS (vehicle control) or combination treatment. Data represent the mean ± standard deviation of triplicates or otherwise stated. *P* values determined by unpaired two-tailed t test; *P < 0.05; ns, not significant.
