# Supplementary material for: Enhancing the efficacy of B7-H3-targeting antibody-drug conjugate DS-7300a in TP53-deficient tumors by inducing ferroptosis
Source: Oncogene. 2026 Jul 27;45(36):3753–64. doi: 10.1038/s41388-026-03921-7 (PMC13529587; doi:10.1038/s41388-026-03921-7)
Supplement: Supplementary file 2 [file 41388_2026_3921_MOESM2_ESM.pdf]

Figure 1:

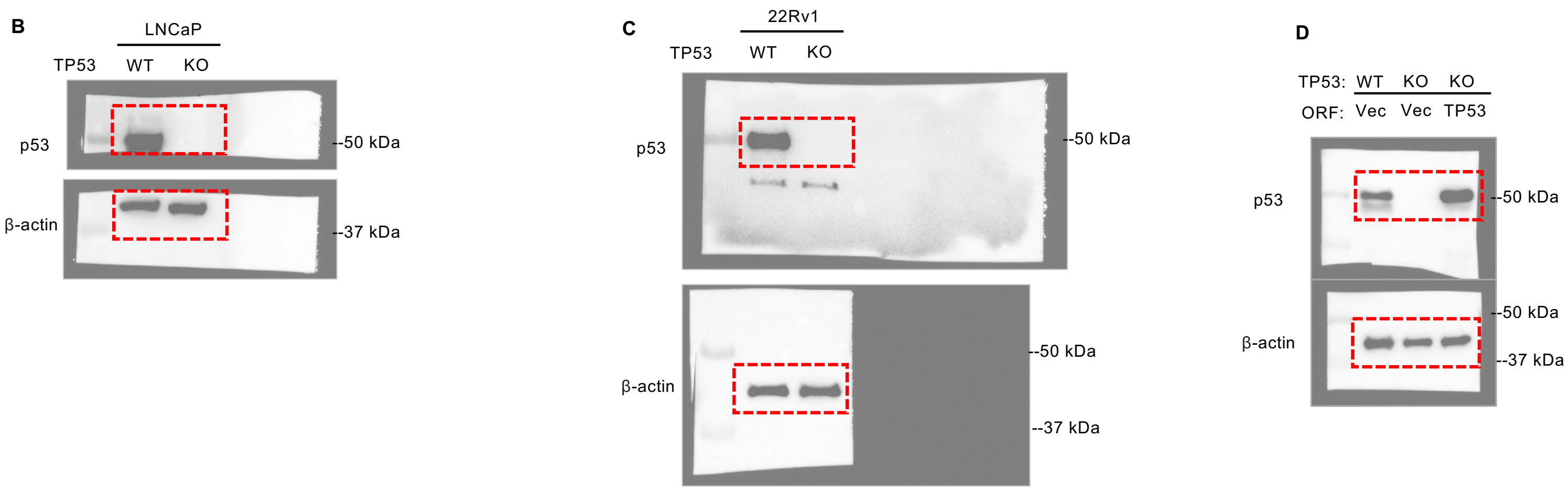
B
LNCaP
TP53
WT
KO
p53
--50 kDa
β-actin
--37 kDa
C
22Rv1
TP53
WT
KO
p53
--50 kDa
β-actin
--50 kDa
--37 kDa
D
TP53:
WT
KO
KO
ORF:
Vec
Vec
TP53
p53
--50 kDa
β-actin
--50 kDa
--37 kDa

Figure 3:

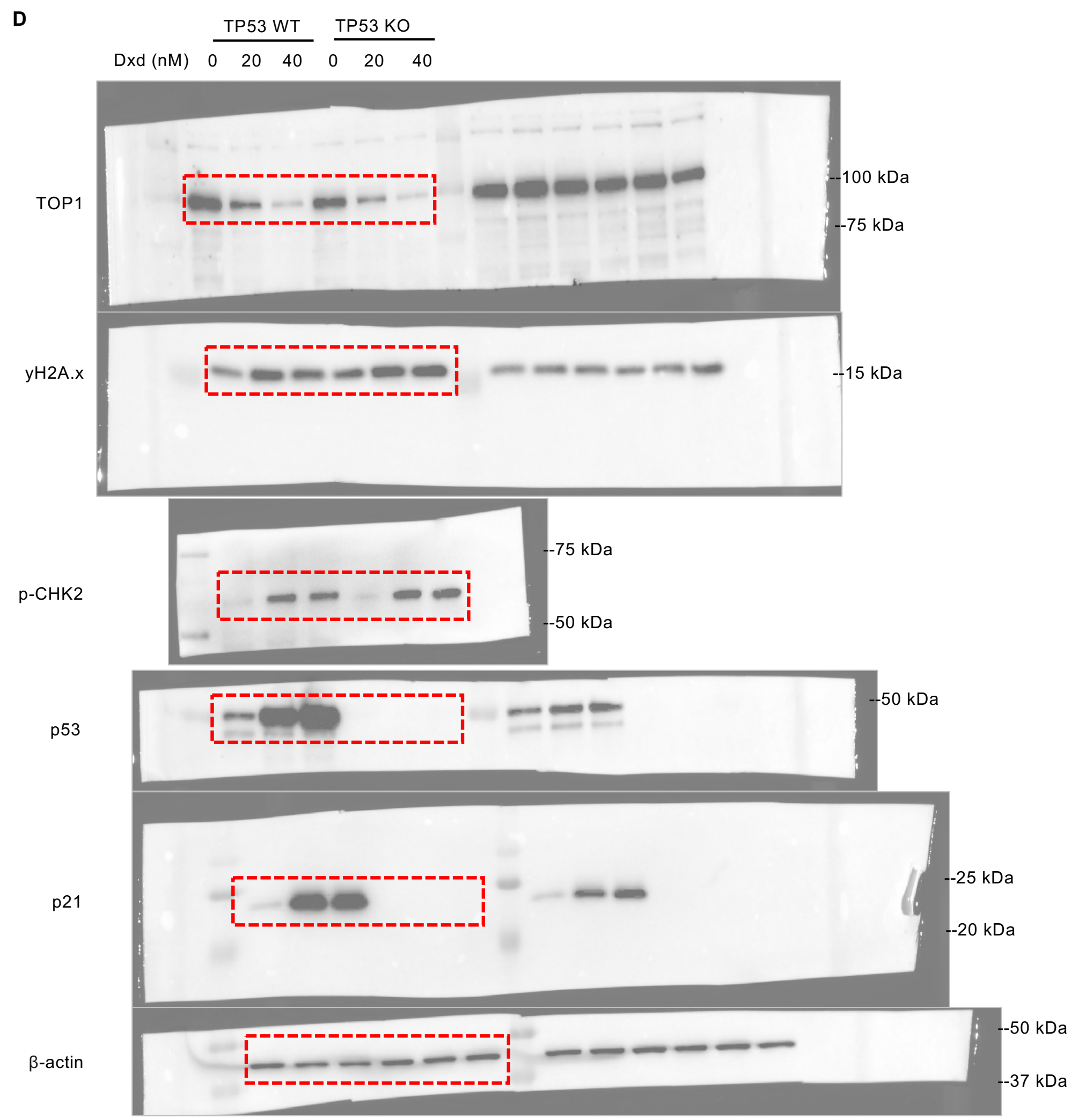
D
TP53 WT
TP53 KO
Dxd (nM) 0 20 40 0 20 40
TOP1
-100 kDa
-75 kDa
yH2A.x
-15 kDa
p-CHK2
-75 kDa
-50 kDa
p53
-50 kDa
p21
-25 kDa
-20 kDa
β-actin
-50 kDa
-37 kDa

Figure 4:

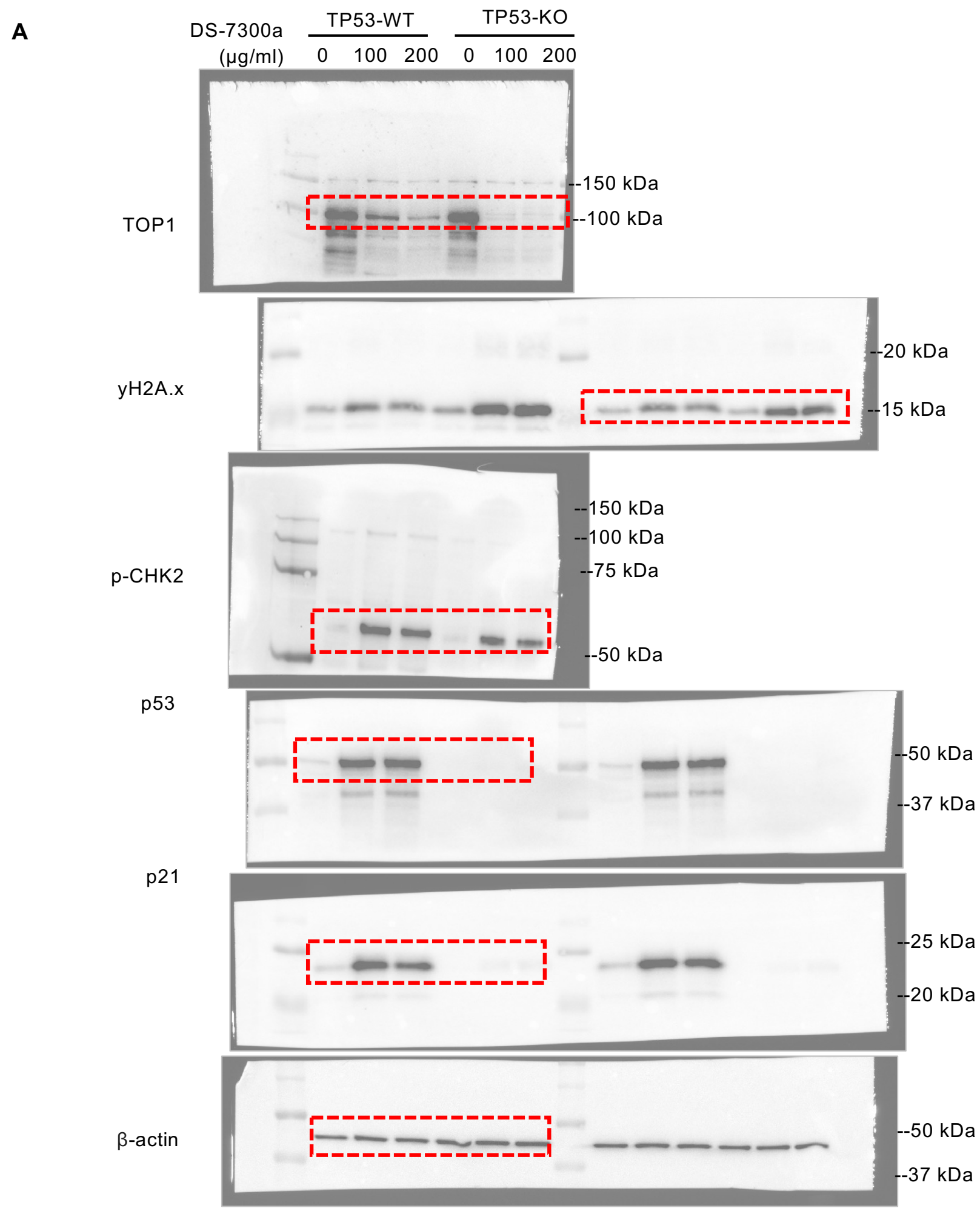

A
DS-7300a
(µg/ml)
TP53-WT
TP53-KO
0 100 200 0 100 200
TOP1
-150 kDa
-100 kDa
yH2A.x
-20 kDa
-15 kDa
p-CHK2
-150 kDa
-100 kDa
-75 kDa
-50 kDa
p53
-50 kDa
-37 kDa
p21
-25 kDa
-20 kDa
β-actin
-50 kDa
-37 kDa

Figure 5:

A

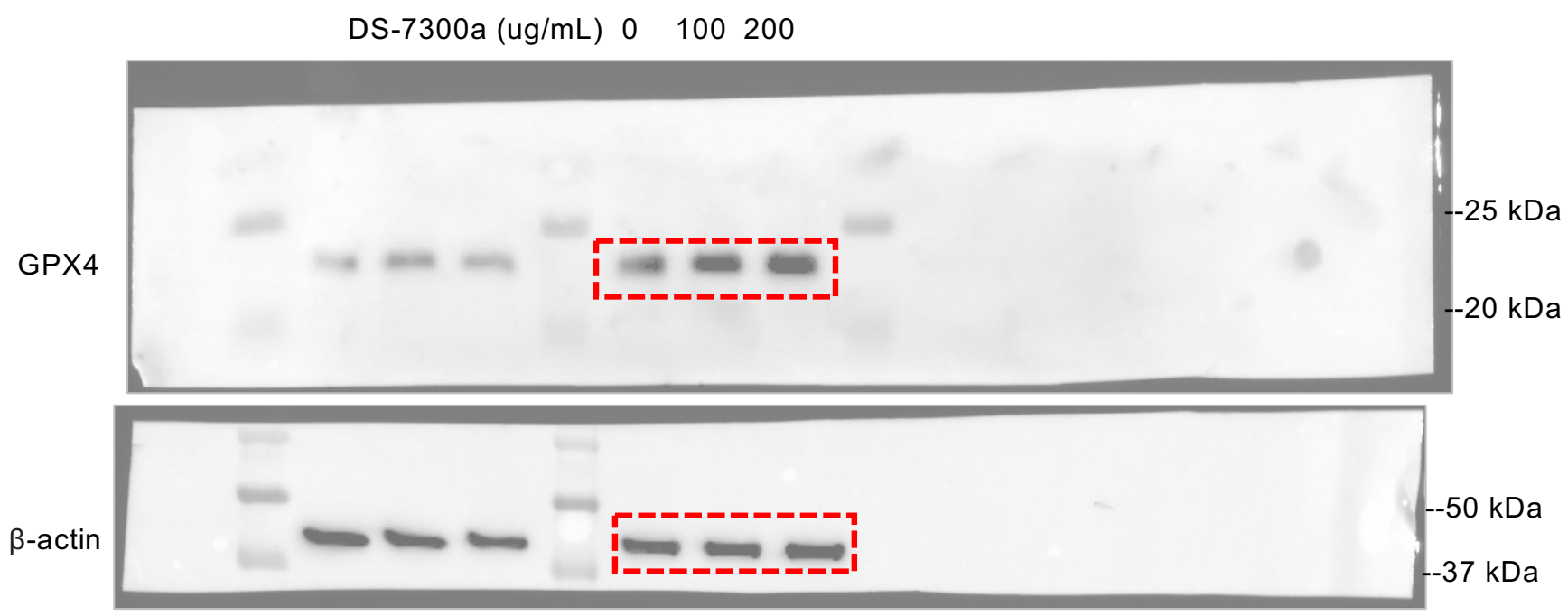
DS-7300a (ug/mL) 0 100 200
GPX4
β-actin
--25 kDa
--20 kDa
--50 kDa
--37 kDa

B

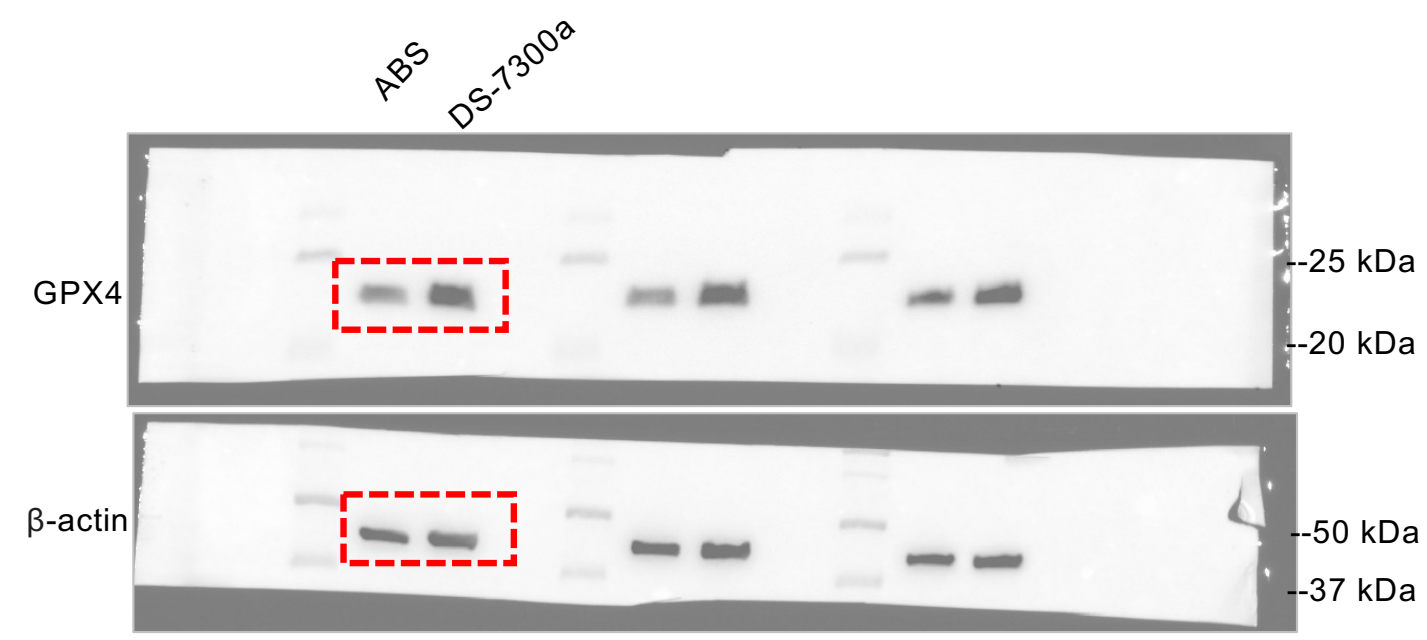
ABS
DS-7300a
GPX4
β-actin
--25 kDa
--20 kDa
--50 kDa
--37 kDa

Figure S1:

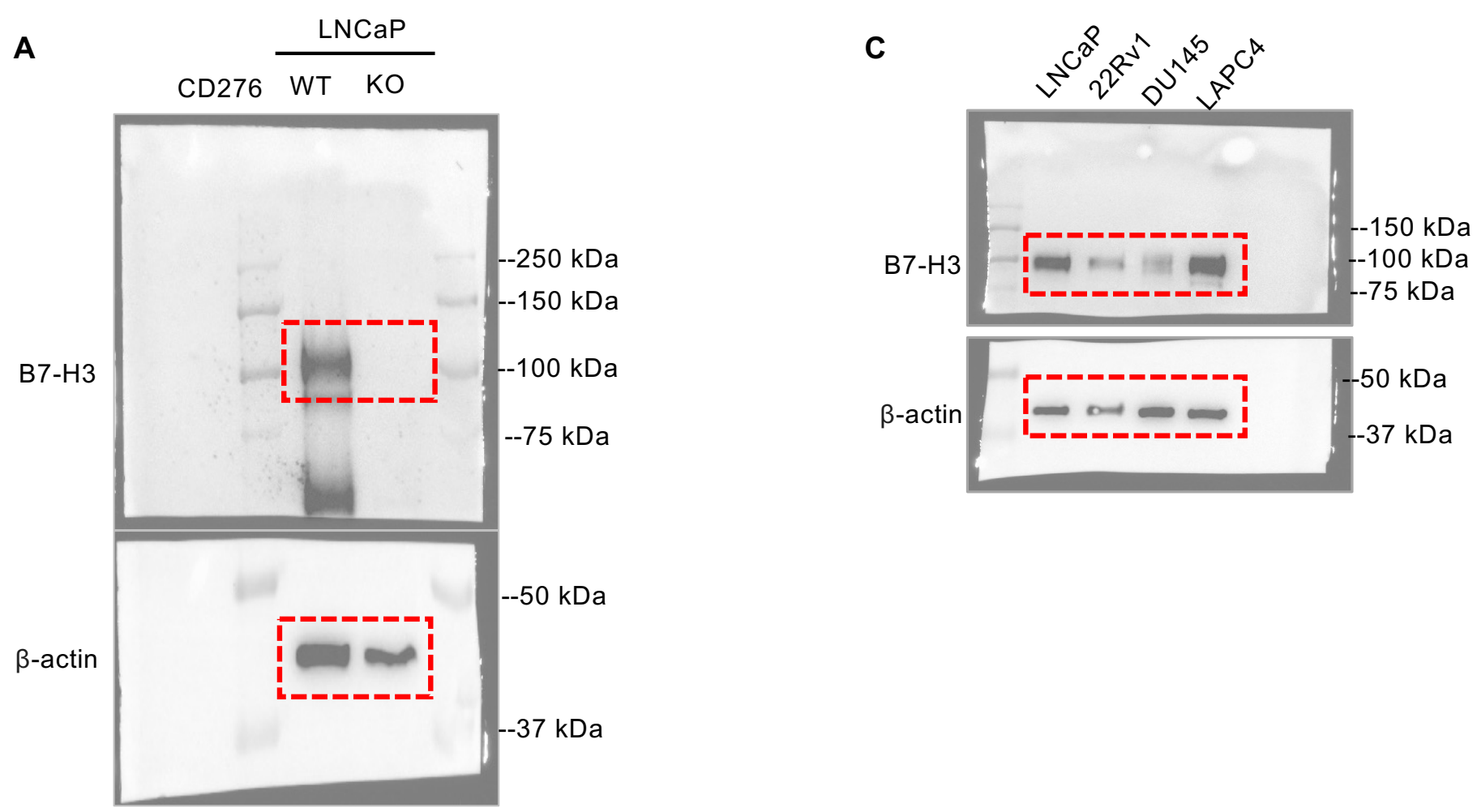
A
LNCaP
CD276
WT
KO
B7-H3
β-actin
--250 kDa
--150 kDa
--100 kDa
--75 kDa
--50 kDa
--37 kDa
C
LNCaP
22Rv1
DU145
LAPC4
B7-H3
β-actin
--150 kDa
--100 kDa
--75 kDa
--50 kDa
--37 kDa

Figure S3:

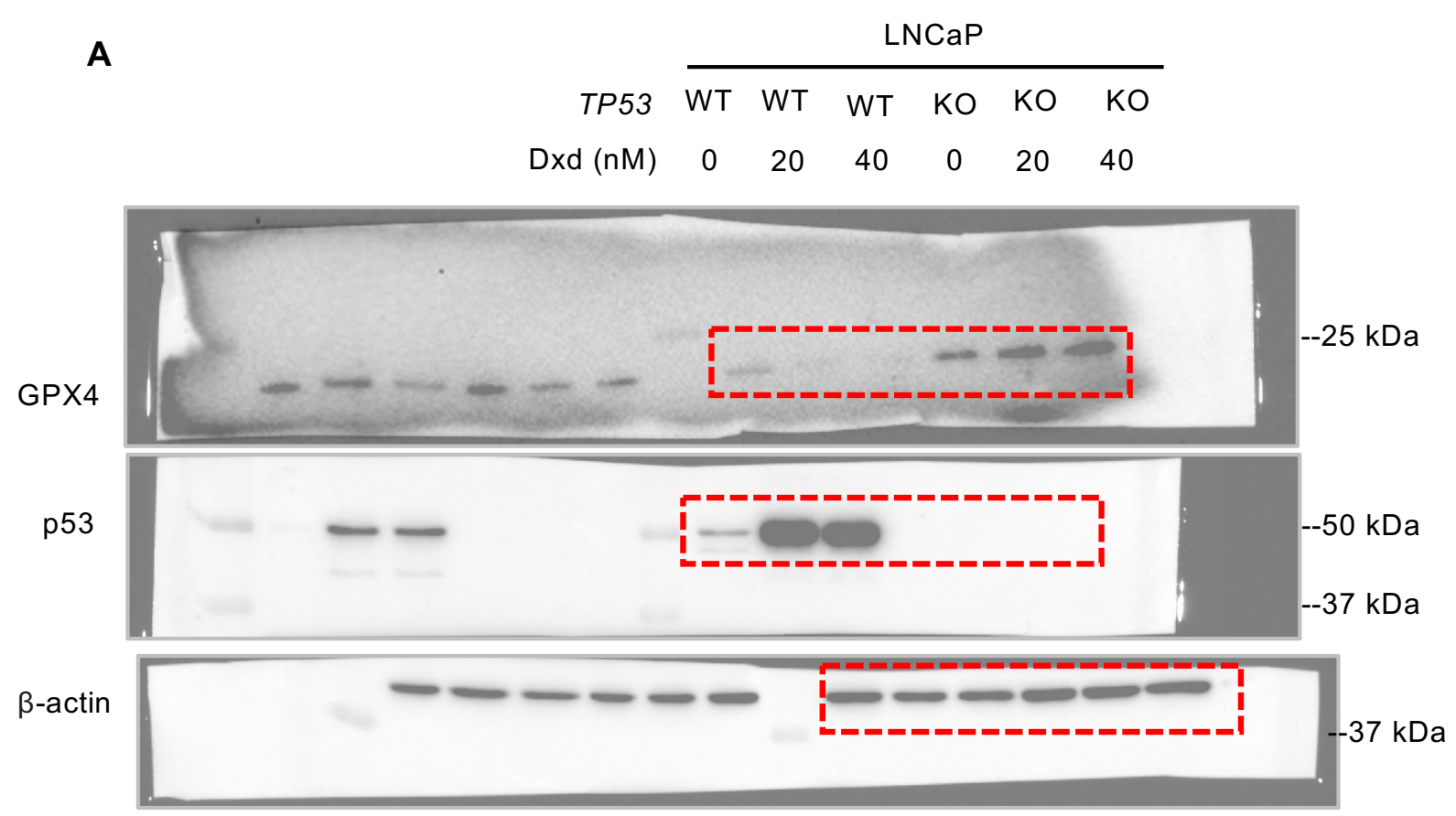
A
LNCaP
TP53
WT
WT
WT
KO
KO
KO
Dxd (nM)
0
20
40
0
20
40
GPX4
--25 kDa
p53
--50 kDa
--37 kDa
β-actin
--37 kDa
